# Retraction Note to: Intra-articular injection in the knee of adipose derived stromal cells (stromal vascular fraction) and platelet rich plasma for osteoarthritis

**DOI:** 10.1186/s12967-021-02852-z

**Published:** 2021-04-26

**Authors:** Himanshu Bansal, Kristin Comella, Jerry Leon, Poonam Verma, Diwaker Agrawal, Prasad Koka, Thomas Ichim

**Affiliations:** 1 RegennMed Research and Therapeutics LLP, Chattarpur, Delhi India; 2 US Stem Cell, Inc, Sunrise, FL USA; 3 Advance Health Institute Mayaguez, Puerto Rico, USA; 4 Mercy Medical Centre, Roseburg, OR USA; 5 Department of Virology and Immunology, Hafkine Institute, Mumbai, Maharashtra 400012 India; 6 Regenerative Medicine Institute, Tijuana, Mexico; 7 Mother Cell Spinal Injury & Stem Cell Research, Anupam Hospital, Second Floor, Kashipur Bypass Road, Rudrapur, Uttarakhand 263153 India

## Retraction Note to: J Transl Med (2017) 15:141 10.1186/s12967-017-1242-4

The Editor-in-Chief has retracted this article [1] due to a number of concerns that have come to light after publication. The clinical trial registry [2] for this study states that the study start date is 12 February 2012. However, documentation regarding the ethical approval provided by the corresponding author shows that the authors applied for ethics approval on 18 February 2012, and that the ethics committee approved the study on 17 September 2012. Additionally, the time stamp on Fig. 4a is 11 September 2012 and on Fig. 4b it’s 27 February 2010, which suggests the research was conducted before ethics approval was obtained. The Editor-in-Chief therefore no longer has confidence in the reliability of the data reported in the article.

Prasad Koka agrees to this retraction. Himanshu Bansal, Kristin Comella and Thomas Ichim do not agree to this retraction. Jerry Leon, Poonam Verma and Diwaker Agrawal have not responded to any correspondence from the editor/publisher about this retraction.
